# The FT4/FT3 ratio combined with thyroglobulin and parathyroid hormone for distinguishing benign from malignant thyroid nodules: a retrospective study based on histopathological diagnosis

**DOI:** 10.3389/fmed.2026.1899842

**Published:** 2026-09-03

**Authors:** Zhulin Zhang, Yun Lan

**Affiliations:** Department of Clinical Laboratory, School of Medicine, Nanjing Tongren Hospital, Southeast University, Nanjing, Jiangsu, China

**Keywords:** FT4/FT3 ratio, malignant thyroid tumor, multivariate logistic regression, parathyroid hormone, ROC analysis, thyroglobulin, thyroid nodules

## Abstract

**Purpose:**

To evaluate the diagnostic value of the FT4/FT3 ratio combined with other laboratory markers in distinguishing malignant from benign thyroid nodules.

**Methods:**

This retrospective study analyzed 359 patients with thyroid nodules (142 benign, 217 malignant) based on histopathological results. Clinical and laboratory data, including FT3, FT4, FT4/FT3 ratio, thyroglobulin (TG), parathyroid hormone (PTH), and TSH, were collected. Statistical analyses included ROC curves and multivariate logistic regression.

**Results:**

Malignant nodules showed higher FT4/FT3 ratios, TG, PTH, and TSH levels (*p* < 0.001). The FT4/FT3 ratio demonstrated moderate diagnostic accuracy (AUC = 0.708). TG and PTH had higher AUCs (0.750 and 0.776, respectively). Combining these markers improved the AUC to 0.896. Higher FT4/FT3 ratios, TG, and PTH were independent malignancy risk factors (*p* < 0.05).

**Conclusion:**

The FT4/FT3 ratio, combined with TG and PTH levels, enhances the differentiation of malignant thyroid nodules, offering a reliable diagnostic tool.

## 1 Background

In the realm of endocrinology, thyroid cancer has become a significant global health concern, with its incidence rates escalating over the past few decades (1, 2). While advancements in diagnostic technologies have been significant, the challenge of distinguishing malignant thyroid tumors from benign nodules at an early stage remains a critical hurdle in clinical settings (3–5). The introduction and integration of biomarkers into the diagnostic process have been transformative, opening new pathways for tailored approaches in the treatment and management of thyroid cancer, embodying the essence of precision medicine in this specialized field. The thyroid gland, an essential player in the endocrine system, orchestrates metabolic processes through its hormone production. The occurrence of thyroid nodules is a frequent scenario in medical examinations, presenting complex puzzle due to their diverse origins, which include both benign and malignant conditions (6). Conventional diagnostic approaches like ultrasonography and fine-needle aspiration cytology (FNAC), despite their utility, have their limitations. Although FNAC is a widely recognized diagnostic procedure for thyroid pathologies, it has inherent limitations, including yielding indeterminate results in 20-30% of cases, necessitating thyroid surgery for a definitive diagnosis (7). Moreover, studies have shown varying degrees of sensitivity, specificity, and accuracy in FNAC, highlighting its limitations in definitive cancer diagnosis and the potential for misdiagnosis (8–11).

In the exploration of thyroid physiology and pathology, thyroglobulin (TG) and parathyroid hormone (PTH) have emerged as significant markers. TG, exclusive to thyroid tissue, is integral to the production and storage of thyroid hormones (12). While TG is primarily established for postoperative surveillance of differentiated thyroid cancer, recent studies have suggested its potential utility in pre-operative risk stratification, as elevated TG levels may reflect increased tumor burden and metabolic activity (13–15). PTH, emanating from the parathyroid glands, is vital for maintaining calcium balance within the body. Although PTH is not a thyroid-specific biomarker, emerging evidence suggests that thyroid malignancies may influence calcium metabolism and parathyroid function through local invasion, surgical trauma, or paraneoplastic mechanisms (16, 17). Fluctuations in the levels of these markers have been linked to various thyroid disorders, prompting a closer examination of their roles in thyroid cancer (18). The FT4/FT3 ratio, a critical metric, sheds light on the intricate balance between triiodothyronine (T3) and thyroxine (T4), offering a window into the functional status of the thyroid gland and its hormone synthesis mechanisms (19, 20). Deviations in this ratio may signal the presence of malignancies or other thyroid dysfunctions.

This study conducts a thorough retrospective examination of a considerable patient cohort from Nanjing Tongren Hospital, School of Medicine, Southeast University, employing these biomarkers to decipher their utility in differentiating thyroid cancers. This research integrates a spectrum of clinical data, biochemical markers, and sophisticated statistical analyses to refine the diagnostic framework for identifying malignant thyroid tumors. The objective is to enhance the precision of early detection methods and contribute to the strategic management of thyroid cancer.

## 2 Methods

### 2.1 Study cohort and design

In this research, we retrospectively analyzed the records of 373 individuals diagnosed with thyroid nodules at Nanjing Tongren Hospital, School of Medicine, Southeast University from August 2018 to December 2023. The selection criteria included: (1) fulfilling the diagnostic benchmarks for thyroid tumors; (2) patient ages spanning 18 to 75 years. The exclusion criteria encompassed: (1) individuals with prior or concurrent malignant tumors; (2) subjects diagnosed with additional thyroid conditions such as hyperthyroidism, hypothyroidism, or Hashimoto's thyroiditis; (3) individuals treated with thyroid-specific medications like Euthyrox. Informed consents were obtained from all participants, and the ethics committee sanctioned the study. After applying the criteria, 359 participants were deemed eligible for inclusion, whereas 14 were excluded based on the exclusion criteria: 2 for concurrent malignancies, 4 due to other thyroid conditions, and 8 for lacking comprehensive data. Participants were subsequently categorized into benign (*n* = 142) and malignant (*n* = 217) thyroid nodule groups according to their final histopathological diagnosis based on postoperative pathology reports. All diagnoses were confirmed by pathological examination of surgically resected thyroid specimens, with benign nodules confirmed as non-malignant and malignant nodules confirmed as thyroid carcinoma.

### 2.2 Data collection

Data were collected from medical records, including gender, age, and several biomarkers such as thyroglobulin (TG), parathyroid hormone (PTH), free triiodothyronine (FT3), free thyroxine (FT4), and the FT4/FT3 ratio. Thyroid-stimulating hormone (TSH), total protein (TP), albumin (ALB), alanine aminotransferase (ALT), aspartate aminotransferase (AST), creatinine (CRE), total cholesterol (CHOL), triglycerides (TG1), high-density lipoprotein (HDL), and low-density lipoprotein (LDL) were also examined. Additionally, pulse (P), respiration (R), systolic blood pressure (SBP), diastolic blood pressure (DBP), and the presence of diabetes mellitus (DM), hypertension, and other nodules were assessed. All blood samples were collected preoperatively, prior to any biopsy or surgical intervention, under standardized fasting conditions. Laboratory measurements were performed using commercially available immunoassay kits on the same platform (Roche Cobas e601) to ensure consistency.

### 2.3 Statistical analyses

Statistical analysis was conducted using SPSS 20.0 software. Normality of continuous variables was assessed using the Shapiro-Wilk test. Variables conforming to a normal distribution were expressed as mean ± SD and compared using the independent samples *t*-test. Variables with non-normal distribution were expressed as median (interquartile range, IQR) and compared using the Mann-Whitney *U*-test. Enumeration data were presented as frequencies and percentages, with the Chi-square (χ^2^) test used for comparisons. Receiver operating characteristic (ROC) curve was implemented to determine the optimal cut-off value and evaluate the differential diagnostic efficacy of different indices. Variables selected for the multivariate logistic regression included TG, PTH, FT4/FT3 ratio, and TSH based on univariate screening (*P* < 0.05) combined with clinical relevance; age, TP, ALB, and T-CHO were included as adjustment variables. The combined score was derived from the multivariate logistic regression model. The predicted probability (logit) from the model was used as the combined diagnostic index, and the ROC curve was generated based on this predicted probability. *P* value < 0.05 was considered statistically significant.

## 3 Results

### 3.1 Comparison of clinical characteristics between the patients with benign and malignant thyroid nodule

In this study, a comprehensive comparison of clinical data between benign (*n* = 142) and malignant thyroid nodule groups (*n* = 217) revealed several significant differences, providing insights into the potential diagnostic value of various laboratory indicators (Table 1). Histopathological classification revealed that papillary thyroid carcinoma (PTC) was the predominant subtype in malignant thyroid nodule groups, accounting for 187 cases (86.2%). Follicular thyroid carcinoma (FTC) was present in 18 cases (8.3%), medullary thyroid carcinoma in 6 cases (2.8%), and other subtypes (including anaplastic and poorly differentiated carcinomas) in 6 cases (2.8%). Age distribution presented a statistically significant difference (*p* = 0.023), with the malignant group having a younger median age (46.50 years) compared to the benign group (51.00 years). Gender distribution showed no significant difference (*p* = 0.061), though males were more represented in the malignant group (34.10%) compared to the benign group (24.65%). Regarding thyroid-related markers, thyroglobulin (TG) levels were significantly elevated in the malignant group (40.61 μg/L) compared to the benign group (14.14 μg/L, *p* < 0.001). Similarly, parathyroid hormone (PTH) levels were higher in the malignant group (42.55 pg/mL) than in the benign group (28.32 pg/mL, *p* < 0.001). The FT4/FT3 ratio demonstrated a notable increase in the malignant group (3.42) vs. the benign group (2.96, *p* < 0.001), indicating its potential diagnostic relevance. Free thyroxine (FT4) levels were also higher in the malignant group, while free triiodothyronine (FT3) levels were lower (*p* < 0.001 for both). Thyroid-stimulating hormone (TSH) levels were significantly elevated in the malignant group (2.03 mIU/L) compared to the benign group (1.57 mIU/L, *p* < 0.001). Serum protein levels, including total protein (TP) and albumin (ALB), displayed significant differences (*p* < 0.001), with TP being lower and ALB slightly higher in the malignant group. Liver function indicators such as alanine aminotransferase (ALT) and aspartate aminotransferase (AST) did not show significant differences (*p* = 0.713 and *p* = 0.112, respectively), nor did triglycerides, high-density lipoprotein (HDL), and low-density lipoprotein (LDL) levels (*p* > 0.05). However, total cholesterol (T-CHO) levels were significantly lower in the malignant group (4.25 mmol/L) than in the benign group (4.72 mmol/L, *p* < 0.001). Creatinine (Cr) levels were slightly higher in the malignant group (*p* = 0.015). Systolic blood pressure (SBP) and diastolic blood pressure (DBP) showed no significant differences between the groups, although DBP was slightly elevated in the malignant group (*p* = 0.023). Lastly, the presence of additional nodules was significantly more frequent in the malignant group (*p* < 0.001).

**Table 1:** Comparison of demographic characteristics between benign and malignant thyroid nodule group.

Clinical data	Benign thyroid nodule group (*n* = 142)	Malignant thyroid nodule group (*n* = 217)	*P*
Gender [*n* (%)]			0.061
Male	35 (24.65)	74 (34.10)	
Female	107 (75.35)	143 (65.90)	
Age (yrs)	51.00 (40.25, 58.00)	46.50 (37.00, 56.00)	0.023
TG (ug/L)	14.14 (4.24, 24.99)	40.61 (12.62, 64.37)	< 0.001
PTH	28.32 (18.93, 37.41)	42.55 (33.30, 55.43)	< 0.001
FT3	4.97 ± 0.67	4.62 ± 0.68	< 0.001
FT4	14.84 (13.51, 16.27)	16.25 (14.21, 18.09)	< 0.001
FT4/FT3	2.96 (2.72, 3.30)	3.42 (3.03, 3.98)	< 0.001
TSH	1.57 (1.04, 2.29)	2.03 (1.26, 3.01)	< 0.001
TP	69.40 (65.38, 72.78)	66.80 (63.50, 70.60)	< 0.001
ALB	44.45 (42.00, 46.48)	43.05 (40.85, 45.73)	< 0.001
ALT	16.00 (12.00, 25.00)	15.00 (11.00, 26.25)	0.713
AST	17.00 (14.00, 20.00)	16.00 (13.00, 20.00)	0.112
CRE	60.40 (54.60, 70.53)	63.35 (56.88, 76.80)	0.015
T-CHO	4.72 (3.91, 5.88)	4.25 (3.72, 4.90)	< 0.001
TG1	1.62 (0.85, 2.52)	1.18 (0.85, 1.78)	0.005
HDL	1.27 (0.93, 1.55)	1.14 (0.97, 1.40)	0.214
LDL	2.69 (2.01, 3.31)	2.50 (2.12, 3.04)	0.252
P	78.00 (72.00, 81.75)	78.00 (73.00, 80.00)	0.614
R	19.00 (18.00, 20.00)	20.00 (20.00, 20.00)	< 0.001
SBP	124.00 (117.00, 136.75)	128.00 (118.00, 140.00)	0.233
DBP	79.00 (72.00, 88.00)	81.00 (75.00, 90.00)	0.023
DM			0.388
Yes	7 (4.93)	17 (7.83)	
No	135 (95.07)	200 (92.17)	
Hypertension			0.343
Yes	24 (16.90)	46 (21.20)	
No	118 (83.10)	171 (78.80)	
Other nodes			< 0.001
Yes	4	34	
No	138	183	

TG, Thyroglobulin; PTH, Parathyroid Hormone; FT3, Free Triiodothyronine; FT4, Free Thyroxine; TSH, Thyroid-Stimulating Hormone; TP, Total Protein; ALB, Albumin; ALT, Alanine Aminotransferase; AST, Aspartate Aminotransferase; CRE, Creatinine; CHOL, Cholesterol; TG1, Triglycerides; HDL, High-Density Lipoprotein; LDL, Low-Density Lipoprotein; SBP, Systolic Blood Pressure; DBP, Diastolic Blood Pressure; DM, Diabetes Mellitus; P, Pulse; R, Respiration.

### 3.2 Differential diagnostic value of FT4/FT4 ratio and other thyroid gland associated indices

**Figure 1:**
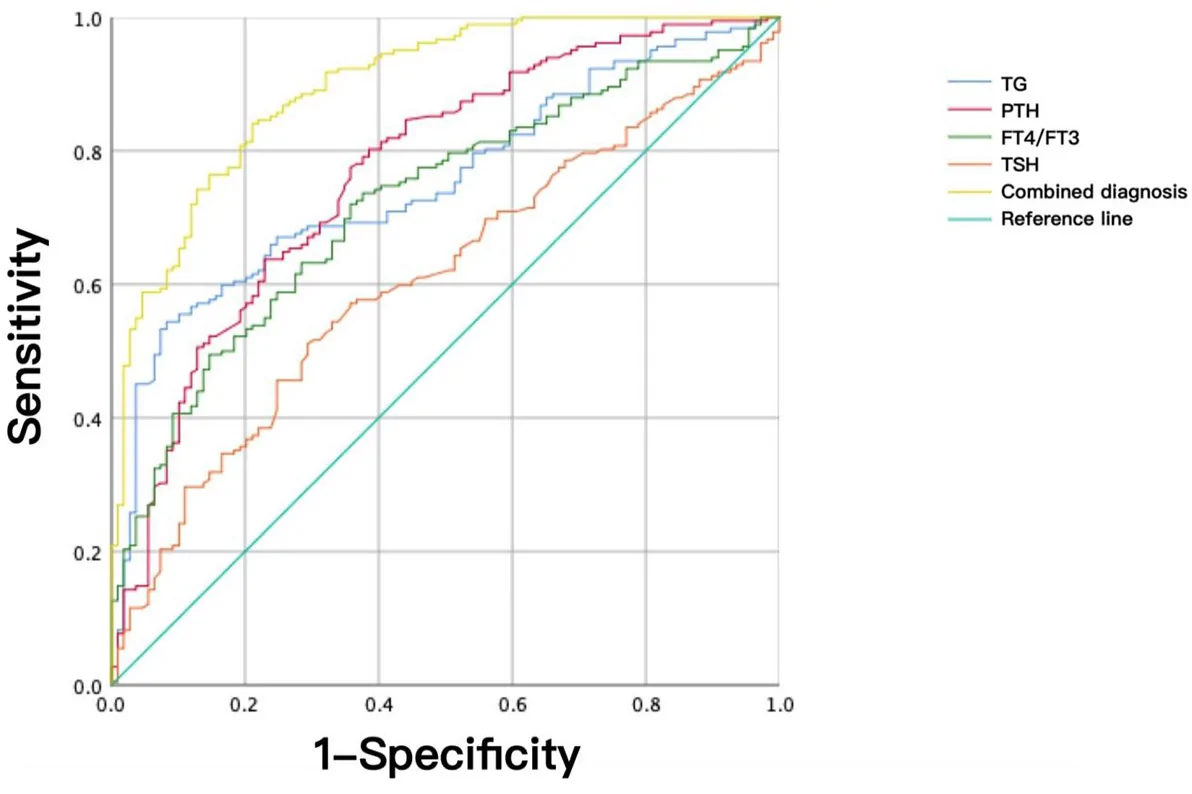
Receiver operating characteristics (ROC) curve of TG, PTH, FT4/FT3 ratio, TSH, and combined diagnosis for differential diagnosis of malignant thyroid nodules.

To evaluate the diagnostic efficacy of various biochemical markers for thyroid cancer, this study implemented receiver operating characteristic (ROC) curves (Table 2). Thyroglobulin (TG) demonstrated significant diagnostic value with an AUC of 0.750 (95% CI: 0.695~0.806), an optimal cut-off value of 38.53 ug/L, a sensitivity of 54.4%, and a specificity of 91.9% (*p* < 0.001, Figure 1). Parathyroid hormone (PTH) also showed high diagnostic accuracy with an AUC of 0.776 (95% CI: 0.722~0.830), an optimal cut-off value of 30.94 pg/mL, a sensitivity of 80.9%, and a specificity of 63.2% (*p* < 0.001, Figure 1). The FT4/FT3 ratio presented an AUC of 0.708 (95% CI: 0.655~0.761), with an optimal cut-off value of 3.07, a sensitivity of 75.1%, and a specificity of 57.7% (*p* < 0.001, Figure 1). Furthermore, a combined diagnostic approach incorporating these indices significantly enhanced the diagnostic accuracy, with an AUC of 0.896 (95% CI: 0.860~0.932), a sensitivity of 84.1%, and a specificity of 78.9% (*p* < 0.001, Figure 1).

**Table 2:** The determination of optimal cut-off value and evaluation of differential diagnostic efficacy of FT4/FT3 ratio and other thyroid associated hormones via ROC curve.

Indices	AUC	95% CI	Optimal cut-off value	Youden index	Sensitivity	Specificity	*P* value
TG	0.75	0.695 0.806	38.53	0.463	54.4	91.9	< 0.001
PTH	0.776	0.722~0.830	30.94	0.441	80.9	63.2	< 0.001
FT4/FT3	0.708	0.655~0.761	3.07	0.328	75.1	57.7	< 0.001
TSH	0.599	0.540~0.658	1.8	0.21	57.6	63.4	0.002
Combined diagnosis	0.896	0.860~0.932		0.63	84.1	78.9	< 0.001

### 3.3 Multi-variate logistic analysis of the influence of FT4/FT4 ratio and other thyroid gland associated indices on the occurrence of malignant thyroid nodule.

Furthermore, the study examined the association between several biomarkers and the risk of thyroid cancer occurrence by multi-variate logistic regression analysis (Table 3). The analysis revealed that elevated levels of these biomarkers were significantly associated with the increased risk of thyroid carcinoma occurrence. For thyroglobulin (TG) levels, the crude odds ratio (OR) was 13.518 (95% CI: 6.44~28.371, *p* < 0.001). After adjustment, the OR remained significant at 13.003 (95% CI: 5.675~29.792, *p* < 0.001), suggesting a strong association even when accounting for potential confounders. Parathyroid hormone (PTH) levels exhibited a crude OR of 15.944 (95% CI: 2.092~121.546, *p* = 0.008). After adjustment, the OR significantly increased to 76.217 (95% CI: 7.699~754.526, *p* < 0.001), underscoring the strong predictive value of PTH in the differential diagnosis of malignant thyroid nodules. The FT4/FT3 ratio had a crude OR of 3.895 (95% CI: 2.477~6.123, *p* < 0.001), and the adjusted OR further increased to 6.329 (95% CI: 3.315~12.084, *p* < 0.05), demonstrating the significant role of the FT4/FT3 ratio in predicting malignant thyroid nodules. Thyroid-stimulating hormone (TSH) levels showed a crude OR of 2.352 (95% CI: 1.522~3.632, *p* < 0.05). After adjustment, the OR was 2.260 (95% CI: 1.226~4.164, *p* < 0.05), maintaining its predictive relevance.

**Table 3:** Multi-variate logistic analysis of the influence of FT4/FT3 ratio and other thyroid associated indices on the differential diagnosis of malignant thyroid nodules.

Indices	Crude		Adjusted	
	OR	P	OR	P
TG ≥ 38.53	13.518 (6.441~28.371)	< 0.001	13.003 (5.675~29.792)	< 0.001
PTH ≥ 30.94	15.944 (2.092~121.546)	0.008	76.217 (7.699~754.526)	< 0.001
FT4/FT3 ≥ 3.07	3.895 (2.477~6.123)	< 0.001	6.329 (3.315~12.084)	< 0.001
TSH ≥ 1.80	2.352 (1.522~3.632)	< 0.001	2.260 (1.226~4.164)	0.009

TG, thyroglobulin; PTH, parathyroid hormone; TSH, thyroid stimulating hormone; FT3, Free Triiodothyronine; FT4, Free Thyroxine.

## 4 Discussion

This study presents a comprehensive analysis of the differential diagnostic value of the FT4/FT3 ratio combined with other laboratory indicators in distinguishing malignant thyroid tumors. The debate in existing literature over the alterations in thyroid hormone levels in thyroid carcinoma is acknowledged, and this research aims to clarify the association between these hormones and thyroid carcinoma (21, 22). It also proposes a cut-off value for the FT4/FT3 ratio, suggesting that values equal to or greater than 3.07 may indicate an elevated risk of cancer. While additional studies are needed to confirm these findings, this research has identified a strong correlation between the proposed quotient and the presence of malignancy. Finally, the significant differences observed in TG, PTH, TSH and the FT4/FT3 ratio between benign and malignant groups align with previous research, underscoring the potential of these markers in thyroid cancer diagnostics.

The increased TG levels observed in malignant thyroid nodules underscores its significance as a biomarker, primarily produced by the thyroid's follicular cells. Thyroglobulin's elevation in the context of thyroid malignancies, especially in differentiated thyroid cancers such as papillary or follicular thyroid cancer, can be attributed to the augmented proliferation and metabolic activity of the tumor cells (13–15). These cells, being more active and numerous in malignant states, produce TG in greater quantities (23, 24). Our study's findings, which highlight the elevated TG levels in patients with thyroid cancer, align with the biomarker's high sensitivity and specificity. This alignment reinforces the established notion that TG serves as a pivotal tool not only for the initial detection of thyroid cancer but also for monitoring disease progression or recurrence. Monitoring TG levels is particularly crucial in the postoperative follow-up of patients who have undergone thyroidectomy, as any rise in TG could potentially indicate a recurrence or the presence of residual thyroid tissue.

Similarly, the elevation of serum PTH levels in the patients with malignant thyroid nodule could suggest involvement of the parathyroid glands or be a compensatory mechanism due to disrupted calcium metabolism, which is often seen in cancer patients (16, 17). Among the biomarkers evaluated, PTH demonstrated the strongest diagnostic performance (AUC = 0.776), suggesting its potential utility as a promising adjunctive marker in thyroid nodule evaluation. Although PTH is typically not associated with thyroid function, its increased levels in individuals with thyroid cancer might reflect a broader systemic reaction to the presence of a tumor or a paraneoplastic syndrome (25). However, PTH is not a thyroid-specific biomarker, and its elevation may be influenced by parathyroid gland dysfunction, vitamin D status, renal function, and calcium metabolism, which were not fully controlled for in this retrospective analysis. Second, the adjusted OR for PTH showed a very wide confidence interval, indicating limited precision. Third, the optimal cut-off value of 30.94 pg/mL derived from this cohort requires external validation. Therefore, while PTH may serve as a useful adjunct in the diagnostic workup of thyroid nodules, particularly when combined with other markers, it should not be used as a standalone diagnostic tool in routine clinical practice without further confirmatory studies.

The diagnostic significance of the FT4/FT3 ratio in the context of thyroid cancer is indeed compelling, although the mechanistic interpretation requires caution. An altered ratio in patients with malignant thyroid conditions points toward an interruption in the typical synthesis and metabolism of thyroid hormones (26, 27). This disruption is likely a consequence of the tumor's interference with the intricate regulatory mechanisms governing thyroid hormone production and release (28, 29). However, it is important to acknowledge that the FT4/FT3 ratio may also be influenced by factors not directly related to thyroid malignancy, including peripheral deiodinase activity, systemic illness, nutritional status, and medications that affect thyroid hormone metabolism. Therefore, the observed difference, while statistically significant, should be interpreted with these potential confounders in mind. Although the FT4/FT3 ratio is presented as a principal biomarker, its individual diagnostic performance (AUC =0.708) was modest compared to TG (AUC = 0.750) and PTH (AUC = 0.776). The FT4/FT3 ratio was selected as the main focus because it represents a novel, easily calculable index derived from two routinely available laboratory tests (FT4 and FT3) that are part of standard thyroid function panels. Its potential value lies not in its individual performance but in its ability to provide complementary information when combined with other markers, as evidenced by the improved combined (AUC =0.896). The integration of a simple ratio such as FT4/FT3 into a diagnostic model alongside other markers makes the approach more accessible in clinical settings without requiring specialized or expensive assays.

In the realm of thyroid physiology, the balance between FT4 (free thyroxine) and FT3 (free triiodothyronine) is crucial. FT4 is generally considered a storage hormone that gets converted into the more active FT3, a process predominantly occurring in peripheral tissues (30, 31). The FT4/FT3 ratio can, therefore, provide insights into the metabolic state and functional well-being of the thyroid gland. In malignant cases, the tumor might alter this conversion process or directly affect the secretion levels of these hormones, thereby skewing the FT4/FT3 ratio. A study highlighted the relationship between the FT4/FT3 ratio and thyroid cancer risk. It found that a higher FT4/FT3 ratio, specifically greater than 3.3, was associated with an increased risk of malignancy in thyroid nodules, indicating that the FT4/FT3 ratio could be a useful marker in evaluating the risk of thyroid cancer (15). In another study, the associations between serum levels of thyroid hormones (fT3, fT4, FT4/FT3 ratio) and thyrotropin (TSH) with the prognosis of PTC were examined (14). The study found that high FT4/FT3 ratio and TSH levels were associated with a higher risk of recurrence (14). This suggests that these markers, particularly the FT4/FT3 ratio, could be useful in predicting the recurrence of PTC and thus assist in tailoring more effective treatment plans. Our study's logistic regression analysis, which revealed a significant correlation between the FT4/FT3 ratio and the risk of thyroid malignancy, underscores the potential of this ratio as a biomarker. This association suggests that thyroid cancer could lead to distinctive alterations in hormone synthesis or secretion, reflective of the tumor's biological behavior and its impact on thyroid function. Moreover, understanding these hormonal changes has clinical implications. For instance, an abnormal FT4/FT3 ratio could aid in differentiating between benign and malignant thyroid nodules, thereby guiding clinical decision-making regarding further diagnostic evaluations or interventions. It also might have prognostic value, as the degree of hormonal disruption could relate to the tumor's aggressiveness or stage.

It should be noted that the adjusted odds ratio for PTH (OR = 76.217, 95% CI: 7.699–754.526) showed a very wide confidence interval, suggesting limited precision and possible model instability. This may be due to the relatively small number of patients with PTH values above the cut-off in the benign group (approximately 20% of benign cases had PTH >30.94 pg/mL), which reduced the statistical power for this comparison. Additionally, PTH is not a thyroid-specific marker, and its elevation in thyroid malignancy may be influenced by various factors including local parathyroid gland involvement, calcium metabolism changes, or systemic effects. Therefore, while PTH demonstrated strong diagnostic performance in this cohort, its routine use as a standalone biomarker for thyroid malignancy requires further validation in larger, independent cohorts. The combined diagnostic approach, integrating these markers, yielded an AUC of 0.896, demonstrating exceptional efficacy in distinguishing between benign and malignant thyroid nodules. This suggests that a multifactorial analysis, rather than reliance on a single marker, enhances diagnostic accuracy, aligning with the trend toward comprehensive diagnostic strategies in precision medicine (32, 33).

However, the study has limitations. The retrospective design may introduce selection bias, and the single-center nature of the study might limit the generalizability of the findings. Additionally, while the biomarkers showed significant diagnostic value, their pathophysiological roles in thyroid cancer development remain to be fully elucidated. Future studies should aim to clarify these mechanisms and validate the findings in prospective, multicenter cohorts. Additionally, the malignant cohort was predominantly composed of papillary thyroid carcinoma (86.2%), with limited representation of follicular, medullary, and anaplastic subtypes. Given that different histopathological subtypes may exhibit distinct laboratory profiles, the generalizability of our findings to non-papillary thyroid malignancies is uncertain. Future studies with larger and more balanced subtype representation are needed to validate these findings across the full spectrum of thyroid cancers.

In conclusion, our study underscores the utility of the FT4/FT3 ratio, TG, and PTH as significant biomarkers in the differential diagnosis of thyroid cancer. These findings enhance our understanding of thyroid cancer pathophysiology and offer a promising avenue for improving diagnostic precision, ultimately facilitating early intervention and personalized patient care.

## Data Availability

The original contributions presented in the study are included in the article/supplementary material, further inquiries can be directed to the corresponding author.
